# Frequent Occupational Exposure to *Fusarium* Mycotoxins of Workers in the Swiss Grain Industry

**DOI:** 10.3390/toxins8120370

**Published:** 2016-12-12

**Authors:** Hélène Niculita-Hirzel, Gregoire Hantier, Ferdinand Storti, Gregory Plateel, Thierry Roger

**Affiliations:** 1 Service of Occupational Hygiene, Institute for Work and Health (IST), University of Lausanne and Geneva, 1066 Epalinges-Lausanne, Switzerland; gregoire.hantier@chuv.ch (G.H.); ferdinand.storti@hospvd.ch (F.S.); gregory.plateel@hospvd.ch (G.P.); 2 Infectious Diseases Service, Lausanne University Hospital, 1066 Epalinges-Lausanne, Switzerland; thierry.roger@chuv.ch

**Keywords:** mycotoxins, exposure assessment, aerosols, grain dust, wheat, LC-MS/MS

## Abstract

Type B trichotecens such as deoxynivalenol (DON), 3-acetyldeoxynivalenol (3-ADON), 15-acetyldeoxynivalenol (15-ADON), nivalenol (NIV) and zearalenone (ZEN) are mycotoxins contaminating wheat and wheat dust. Mycotoxins are toxic upon ingestion and considered potentially toxic when inhaled. Whereas dietary exposure to mycotoxins is controlled in food, data on occupational exposure by inhalation by grain workers are scarce. The objectives of this study were to determine the incidence of DON, 3-ADON, 15-ADON, NIV and ZEN in aerosols generated during grain harvesting and unloading and the risk of exposure of grain workers. Aerosols were collected during the threshing of 78 winter wheat fields and grain unloading of 59 grain lots in six grain terminals in the Vaud region (Switzerland). The samples represented the diversity of the winter wheat cultivar and of the farming system (88 treated with fungicides, 46 untreated). Using a HPLC MS/MS method developed to quantify mycotoxins in aerosols, we report that the mycotoxin content of aerosols was not affected by the wheat cultivars or farming system, but that the incidence of the mycotoxins differed between activities. While wheat harvesting generated on average 28, 20 and 1 ng·m^−3^ of DON, NIV and ZEN, respectively, grain unloading generated 53, 46 and 4 ng·m^−3^. Personal sampling revealed that working in a cab was an efficient protective measure. However, it was not sufficient to avoid chronic exposure to multiple mycotoxins. The most exposed activity was the cleaning, exposing workers to DON, NIV and ZEN at concentrations as high as 65, 59 and 3 ng·m^−3^. These data provide valuable information for future studies of mycotoxin toxicity at relevant concentrations on respiratory health.

## 1. Introduction

Chronic exposure of grain workers to grain dust occurs mainly during grain handling and is associated with respiratory symptoms commonly related to chronic bronchitis and asthma-like disorder and the accelerated decline of lung functions [1,2]. Grain dust is a complex mixture of fungal particles, bacteria, insect compounds, animal wastes, inorganic compounds/silicates, chemicals, gases and fumes. While exposure to some of these components was recently reported [3], exposure to grain dust toxins has not been explored.

Serious consideration has been given to toxins—so called mycotoxins—produced by fungi in wheat as one of the causative agents of intoxication by ingestion. The mycotoxins of main concern are deoxynivalenol (DON) and zearalenone (ZEN). These mycotoxins are produced in variable quantities in the plant by *Fusarium* species—mainly *F. graminearum* and *F. culmorum*, depending on the wheat cultivar [4]. The accumulation of mycotoxins in the plant is favored by particular meteorological conditions during wheat flowering, the culture of maize as a previous crop, and reduced tillage [5,6]. However, even when using the lowest susceptible wheat variety and intensive mechanical maize residue mulching treatments, DON contaminations are rarely short of the maximum limit of 1.25 mg·kg^−1^ (recommendation by the European Commission Regulation 2007) in unprocessed cereals when wheat following grain maize was sown after minimal tillage [7]. 

The high frequency of mycotoxins in the grain opens the question of the exposure of grain workers to fungal contaminants through dust inhalation. Indeed, there is a good correlation between grain and wheat dust for the presence of DON and ZEN [8,9], as well as of fungi that might produce them between grain dust and aerosols [10]. Although high exposure to grain dust is inevitable during direct handling of grain or straw or during the cleaning of surfaces contaminated by grain dust [1,3], the identification of determinants of high exposure to mycotoxins may help employers to optimize the safety of workstations.

The first aim of the present study was to obtain quantitative data on the occurrence of DON and ZEN as well as of three others frequent mycotoxins of wheat—3 acetyl deoxynivalenol (3-ADON), 15 acetyl deoxynivalenol (15-ADON) and nivalenol (NIV)—in aerosols generated during wheat handling (threshing and unloading) and harvester cleaning. The second aim was to determine the personal exposure of grain workers to mycotoxins in order to estimate the efficiency of the existing protective equipment and to identify the most exposing tasks. As a prerequisite to this study, we developed a HPLC MS/MS method for the quantification of mycotoxins in airborne samples. Wheat grain and aerosols were collected in parallel from the same fields during threshing. The HPLC MS/MS method was applied on small amounts of dust particles separated from wheat grain in the laboratory and then on aerosol samples. This allowed us to study the impact of factors that might affect mycotoxin accumulation in the plant, such as the wheat cultivar, tillage and crop rotation.

## 2. Results

### 2.1. Mycotoxins in Aerosols Generated during Threshing and Grain Unloading 

**Figure 1:**
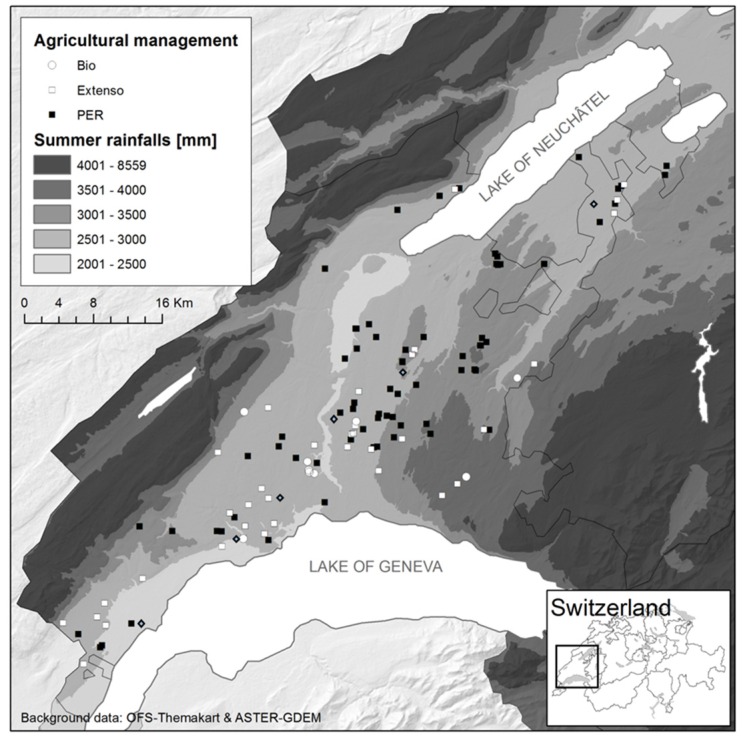
Study area in the granary region of Vaud (Switzerland). The white and black dots correspond to the sampled wheat fields and the blue ones to grain terminals. The farming system used in wheat fields is indicated: white circle for organic farming, white square for extensive farming and black square for conventional farming. The background represents the summer rainfall level.

A total of 137 samples from all over the granary region (560 km^2^) of Vaud (Switzerland) were collected during the threshing of 78 fields and the grain unloading of 59 grain lots in six grain terminals (Figure 1). One hundred and thirty-two samples corresponded to 23 different winter wheat cultivars, three to triticale and two to spelt wheat. The sample collection reflected the diversity of the farming system of the region, coming from 88 conventional, 35 extensive and 11 organic fields.

**Figure 2:**
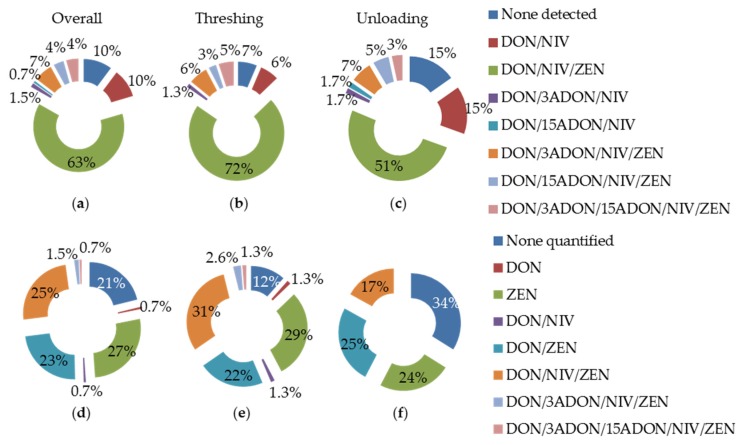
Co-occurrence of mycotoxins in aerosols. (**a**–**c**) The proportions of samples for which the different combinations of mycotoxins were detected in, respectively, overall threshing and grain unloading samples; (**d**–**f**) Samples for which all mycotoxins from a particular combination were detected at a quantifiable level.

A highly sensitive HPLC MS/MS method was developed to detect DON, 3-ADON, 15-ADON, NIV and ZEN in small grain dust samples and, then, in aerosols (see Materials and Methods). The spike recovery was >90% for DON, 15-ADON, NIV and ZEN and 74% for 3-ADON (Table 1). The limit of detection (LOD) and the limit of quantification (LOQ) of DON, 3-ADON, 15-ADON, NIV and ZEN were 0.35 and 1.15, 0.45 and 1.50, 0.66 and 2.21, 0.33 and 1.16 and 0.03 and 0.12 ng·m^−3^, respectively (Table 1). DON and NIV were present above the LOD in the majority (90%) of samples as well as ZEN (77%), while 3-ADON (12%) and 15-ADON (9%) were rarely detected (Table 2). ZEN, NIV, DON, 3-ADON and 15-ADON levels were above the LOQ in 100% (106/106), 50% (61/123), 39% (48/123), 18% (3/17) and 8% (1/12) of positive samples, respectively. Combined analyses revealed that 90% of the samples contained at least two mycotoxins and 79% at least one mycotoxin at a quantifiable level (Figure 2).

The mean concentrations of mycotoxins present over the LOQ in aerosols are presented in Table 2. There was a significant correlation between most mycotoxins in aerosols (Spearman rank coefficient r_s DON-3-ADON_ = 0.3212, r_s DON-NIV_ = 0.8615 r_s DON-ZEN_ = 0.6688 r_s NIV-ZEN_ = 0.6987). The mean concentrations of DON, NIV and ZEN were higher during grain unloading than threshing (Table 2) and reached concentrations as high as 243.9, 297.2 and 15.6 ng·m^−3^, respectively. There was no significant difference in the incidence or on the level of the mycotoxins between cultivars (Table S1), the farming system or the fungicide treatment. Consequently, data were pooled for further analyses.

### 2.2. Comparison of Mycotoxins Content in Aerosols Generated during Threshing and Grain Unloading 

Differences in the risk of exposure to mycotoxins with the type of activity were sought by comparing the incidence and the level of mycotoxins in aerosols collected during threshing and grain unloading. The frequency of samples contaminated with at least one mycotoxin was higher during threshing (93%) than grain unloading (85%) (Figure 2). In particular, ZEN was detected more frequently in the aerosols collected during threshing than during grain unloading (OR 0.38 [0.1203774; 1.202831]; *p* = 0.008; Table 2) while no difference between these two activities was noticed for DON, 3-ADON, 15-ADON and NIV (Table 2). In addition, the risk of exposure to the three mycotoxins DON, NIV and ZEN was much higher during harvesting (72%) than grain unloading (51%) (Figure 2). The concentration of these mycotoxins was significantly higher in the aerosols generated during threshing than during grain unloading (Spearman rank coefficient for DON r_s_ = 0.3149, *p* = 0.0002, for NIV r_s_ = 0.3772, *p* < 10^−4^, and for ZEN r_s_ = 0.3772, *p* < 10^−4^; Table 2). 

### 2.3. Personal Exposure of Grain Workers to Mycotoxins

To estimate the exposure of grain workers to mycotoxins, personal air sampling was conducted for seven harvesters during harvesting and for 12 grain terminal operators during grain unloading. Personal exposure was also estimated during cleaning activities. The mean values obtained are summarized in Table 3. Grain workers were frequently exposed to DON, NIV and ZEN during all grain handling activities. In grain terminals in particular, the frequency of exposure was higher when the operators were in direct contact with the grain (frequent in and out movements between the office and the unloading dock, continuous presence at the terminal; Table 3) than when they were continuously working in the office. The highest levels of exposure to mycotoxins were noticed for the cleaning activity, followed by the reception of wheat grain to the grain terminal. 

## 3. Discussion

*Fusarium* mycotoxins are frequent contaminants of wheat. The consequences on health of their ingestion are well known and tolerable daily intake (TDI) values have been established in Europe (TDI for DON and its acetyl derivates: 1.0 mg·kg^−1^ body weight (bw) per day, EFSA-CONTAM, 2013; TDI for NIV: 1.2 mg·kg^−1^ bw per day, EFSA-CONTAM, 2013; TDI for ZEN: 0.25 mg·kg^−1^ bw per day, EFSA-CONTAM, 2011). However, the impact of the exposure to mycotoxins on respiratory health remains poorly investigated. To address that issue, we studied the environment of a working population most at risk and identified the most exposing scenarios. Our results show that DON, NIV and ZEN are frequent contaminants of aerosols generated during wheat processing. This finding confirms the ubiquitous presence of mycotoxins in wheat dust and grain. Moreover, they point out the frequent risk of exposure of grain workers to multiple mycotoxins during the wheat harvesting period. The collective protection measures (e.g., working in ventilated cabs) were very efficient since they reduced exposure levels 10- to 20-fold, depending on the activities and the mycotoxin considered. However, a majority of operators were frequently in direct contact with wheat dust: the harvesters when they control the threshing quality or clean the machinery and the grain terminal operators when they sample the grain for analysis. The wearing of personal protective equipment during these processes was largely encouraged. Unfortunately, however, in practice, this advice was rarely followed even during the cleaning procedure, the activity with the highest level of exposure to wheat dust and mycotoxins. 

DON, NIV and ZEN were detected in 72% of aerosols generated during threshing all over the Vaud region. Similarly, elevated frequencies of DON and ZEN were recently reported in grains of cereals grown in Poland and Brazil [11,12], which suggests that our findings are not anecdotic. An increase of *Fusarium* mycotoxin frequency in grain wheat during the last 20 years was pointed out [12]. This has been linked to more frequent rainfall episodes during wheat flowering, as well as to the fact that the prevalence of mycotoxins seems to be higher in grain dust than in grain [13]. Thus, the risk of exposure of grain workers to *Fusarium* mycotoxins is frequent and constant through the years. Another common point between our study and previous ones conducted on grain is the co-occurrence of two or more mycotoxins in most contaminated samples [11,12]. The detection of a single mycotoxin was a rare event (e.g., not observed in the present study, 6% in Bryla et al. [11]) when samples were tested for multiple mycotoxins. Thus, the frequent risk of exposure of grain workers to multiple mycotoxins, in particular DON and ZEN, all over the world is of genuine concern.

Not only harvesters, but also operators of grain terminals were confronted with environments contaminated by DON, NIV and ZEN. However, while harvesters were slightly more frequently exposed than terminal operators, the aerosols generated during threshing contained, on average, lower levels of DON, NIV and ZEN than those generated during grain unloading. One possible explanation is that dust generated during threshing is representative of the full plant, whereas dust generated during unloading originates from the grain itself on which *Fusarium* preferentially develops. The co-occurrence of DON, NIV and ZEN in aerosols was in accordance with that observed in wheat dust. A high correlation between DON and NIV has been observed in wheat spikes [4] and was suggested to result from the production of toxins by the same *Fusarium* species [4,14]. Highly significant correlations between DON and ZEN and NIV and ZEN were also found in our study. Multiple infections of wheat spikes by NIV and ZEN producing species such as *F. graminearum* and *F. culmorum*—the two dominant *Fusarium* species infecting wheat—can explain this finding. The high frequency of co-infections of European wheat [15,16] comforts the assumption that grain workers are chronically exposed to multiple mycotoxins.

Harvesters and grain terminal operators were, in general, protected from direct exposure to wheat dust by working in protected areas such as ventilated cabs. Still, they regularly had to survey the grain process. In fact, operators should work only in offices/cabs in order to be exposed to levels of DON and ZEN as low as 2 and 1 ng·m^−3^, levels similar to those previously observed in the air of the workplace [17]. It is important to point out that this situation was rarely observed in the field and that cleaning activities are highly and systematically exposing grain workers to mycotoxins because of their reluctance to wear personal protective equipment during the hottest and the most stressful period of the year. Our studied population is not a unique case. Similar observations have been reported recently in the Norwegian grain industry where cleaning and the controlling process associated with grain elevators have been identified as strong determinants of increased grain dust exposure [3]. Overall, grain worker populations have a similar exposure risk in different countries due to the nature of the processes involved in wheat harvesting, grain cleaning and quality control. Raising grain workers’ awareness to limit the time spent in close contact with wheat dust is critical to guarantee a low level of exposure to *Fusarium* mycotoxins.

A majority of the particles generated during grain handling are small enough (less than 5 μm) to penetrate airways [18]. Additionally, grain workers are potentially exposed to mycotoxins through direct deposits on skin [19] and through ingestion when breathing occurs by mouth [20] and by food intake as in the general population. Modeling this complex exposure should take into consideration mycotoxin bioavailability through different tissues, data on which is not available. The concentration of no toxicological concern (CNTC, i.e., concentrations assumed to pose no hazard to humans) for airborne mycotoxins is 30 ng·m^−3^ [21]. While our data suggest that common exposure to mycotoxins during harvesting and grain unloading are usually below the CNTC, the cumulative dose of mycotoxins may exceed 20 ng·m^−3^ for operators working at the grain terminal. The frequent exposure to multiple mycotoxins, at least to DON and NIV and frequently to DON, NIV and ZEN, might be of concern. Indeed, exposure to mixtures of mycotoxins has a synergetic effect on epithelial cells, affecting cell viability and cytokine production ([19,22], our unpublished data).

The effects of exposure to grain dust on the respiratory health of grain workers have been reported in multiple epidemiological studies. Correlations between respiratory symptoms and exposure to different components of grain dust point out the irritative and toxic effects of grain dust [3]. Exposure to high levels of DON (more than 8 mg·kg^−1^ body weight) was recognized as a probable factor of acute pathologies in humans (Joint Expert Committee for Food Additives 2010). However, low levels of exposure to grain dust might also be detrimental through the combined exposure to multiple mycotoxins. Further studies establishing a direct correlation between personal exposure to mycotoxins at the work place and toxicity for the respiratory tract (e.g., quantification of cytokines in exhaled breath condensates) should be conducted in order to test this hypothesis. A first approach to address that concern would be to characterize the toxic and immunomodulatory effects of DON, NIV and ZEN alone or in combination/mixtures on the reactivity of airway immune and non-immune cells using in vitro air-liquid interface systems and in vivo preclinical animal models.

## 4. Materials and Methods

### 4.1. Study Area and Sampling Strategy

The study was conducted in the Swiss granary region of Vaud, which provides approximately 25% of the internal consummation in wheat and covers a 560 km^2^ area (Figure 1). Harvesters have been followed between 15 July and 9 August 2010 in 78 fields that represent the diversity of farming practices in Vaud (conventional, extensive and organic farming) with respect to wheat cultivars, wheat species (winter wheat, spelt wheat, and triticale), soil types, fertilization, and fungicide treatments. From each field, the aerosols were sampled systematically at the same place on the combine harvester during the overall threshing process. At the end of the process, 1 kg of grain was collected at different places in the grain truck. 

Six grain terminals well geographically distributed throughout the studied area agreed to participate to this study. Full shift personal sampling of airborne dust was conducted on one day for the 12 grain terminal operators working in these sites. In parallel, stationary airborne sampling was conducted during the unloading of each wheat batch on the unloading dock. 

Aerosols were sampled on PTFE membrane filters of 1.0 μm (SKC Inc., Eighty Four, PA, USA) at a flow rate of 2.0 L·min^−1^ using pocket pumps (MSA Escort Elf, Mine Safety Appliance Company, Pittsburgh, PA, USA or SKC pocket pump 210-1002, SKC Inc.) and clear styrene cassettes with three sections (25 mm diameter, SKC Inc.). The temperature, barometric pressure and relative air humidity were measured at each sampling site with a thermo-hygrometer and barometer PCE-THB 40 (PCE Group Iberica, Albacete, Spain).

### 4.2. Sample Preparation and Extraction

Determination of mycotoxins concentrations in aerosols and grain dust were based on solid phase extraction followed by liquid chromatography-tandem mass spectrometry (LC-MS/MS) analysis. Grain dust sample with a particle size of less than 0.125 mm was obtained from 100 g of grain by sieving with a continuous shaking movement for 3 min using a 15 mL Falcon tube equipped with a stainless steel wire sieve mesh with 0.125 mm openings (VWR International, Radnor, PA, USA). Ten mg were used to establish the methodology that was then applied on the overall particles collected on the PTFE filter. The grain dust was recovered from PTFE filter by washing the cassettes with the same solvent as that used to resuspend the grain dust extracted from grain dust: 2 mL methanol:water (70:30, *v*/*v*). Then the samples were sonicated at 20 °C for 15 min. After centrifugation at 4000 rpm for 3 min at 20 °C, the supernatant was transferred in a Vivaspin 4 filter (10 KDa, Satorius Stedium biotech, Goettingen, Germany), re-extracted with 1 mL methanol:water (70:30, *v*/*v*) and filtrated at 4000 rpm for 60 min at 35 °C (Eppendorf 5810R, Eppendorf, Hamburg, Germany). The filtrate was evaporated to dryness in a Turbo Vap^®^ LV (Caliper Life Science, Hopkinton, MA, USA) during 90 min at 60 °C. Finally, the residue was dissolved in 950 μL injection solvent, consisting of methanol:water (80:20) supplemented with 5 μM ammonium acetate and spiked with 50 μL of ^13^C internal standards at 1 μg/mL. 

### 4.3. Regents and Chemicals

The mycotoxin calibration standards DON, NIV, 3-AcDON, 15-AcDON and ZEA (100 ng·μL^−1^ each, quality Oekanal^®^) were purchased from Sigma-Aldrich (Seelze, Germany) and U-[13C15]-DON (25.2 ng·μL^−1^), U-[13C17]-3-AcDON (25 ng·μL^−1^), U-[13C15]-NIV (25.2 ng·μL^−1^), U-[13C18]-ZEN (25.9 ng·μL^−1^) were purchased from Biopure (Tulln, Austria). Water and methanol (LC-MS grade) were obtained from Carlo Erba Reagents (Val De Reuil, France). Ammonium acetate (eluent additive for UHPLC-MS) was purchase from Fluka (Sigma-Aldrich, St. Gallen, Switzerland).

### 4.4. LC-MS/MS Analysis

LC-MS/MS analysis was performed on a Ascensis Express F5 column (L = 100 mm, ID = 3.0 mm, 2.7 μm, Supelco, Bellefonte, PA, USA) with a guard Ascensis Express F5 Guard column (L = 5 mm, ID = 3.0 mm, 2.7 μm, Supelco Inc., Bellefonte, PA, USA) using a HPLC Thermo Fisher Dionex Ultimate^®^ 3000 system (Thermo Fisher Scientific Inc., Sunnyvale, CA, USA) consisting of a quaternary pump (Ultimate 3000 pump) and autosampler (Ultimate 3000 autosampler) and thermostating fixed at 40 °C (Ultimate 3000 column compartment). Ten μL of each sample, including ^13^C internal standards were loaded. Chromatographic separation was achieved with a mobile phase consisting of 5 μM ammonium acetate in water (eluent A) and methanol (eluent B) at a flow rate of 400 μL·min^−1^. The following gradient of eluent B was used: starting at 20%, at 20% for 5 min, from 20% to 96% for 14 min, constant at 96% for 20.5 min, from 96% down to 20% for 21 min, constant at 20% for 26 min.

The column effluent was transferred into the mass spectrometer Thermo scientific TSQ quantiva MS/MS instrument (Thermo Fisher Scientific Inc., Sunnyvale, CA, USA) equipped with Ion Max NG electrospray ionization (ESI). The interface was operated in negative ion mode. The vaporization temperature was maintained at 250 °C, the ion spray voltage at −2.8 kV and ion transfer tube at 300 °C. The gas set values were as follows: sheath gas flow 50 arb, auxiliary gas 13 arb and collision gas flow 1.5 m Toor. Quantitative analysis was performed using tandem MS in multiple reaction monitoring mode alternating two transition reaction for each mycotoxin (Table S2) Data processing was carried out using the Thermo Scientific™ Dionex™ Chromeleon™ 7.2 (Thermo Fisher Scientific Inc, Sunnyvale, CA, USA). 

### 4.5. Validation Method

Quantification was held with the calibrations of the matching matrix, freshly produced for each batch of samples. Matrix-matched calibration curves were obtained by adding a diluted mixture of the standards and internal standards to the matrix (10 mg of grain dust) at 0, 0.05, 0.1, 0.2, 0.3 and 0.5 μg·mL^−1^. The calibration function is considered acceptable if the bias measurement is lower than 15% for 0.05 μg·mL^−1^ and 10% for 0.1, 0.2, 0.3 and 0.5 μg·mL^−1^. The non-spiked matrix was analyzed in parallel. Each sample was analyzed in triplicate. To determine the recovery rate, 10 mg of grain dust were spiked with mycotoxins solutions at 0.01 ng·μL^−1^, 0.05 ng·μL^−1^ and 0.1 ng·μL^−1^ in duplicate. The recovery rate, limits of detection (LOD) and limits of quantification (LOQ) are indicated in Table 1. LOD and LOQ were calculated based on signal-to-noise (S/N) ratios of 3:1, and 10:1, respectively, as obtained from chromatograms of grain dust extracts used for calibration method.

### 4.6. Statistical Analyses

Only samples contaminated above the LOD level were considered in mycotoxin incidence analyses and those contaminated above the LOQ level were included in calculations of average values. Univariate associations between mycotoxin concentrations and continuous variables were explored using non-parametric methods. Categories were compared by Kruskal-Wallis test for the association between mycotoxin concentration and categorical variables. The statistical analyses were conducted with Stata Statistical Software: Release 14 (StataCorp LP., College Station, TX, USA).

## Figures and Tables

**Table 1:** Recovery rate, limit of detection (LOD) and limit of quantification (LOQ) of DON, 3-ADON, 15-ADON, NIV and ZEN.

Variables	DON	3-ADON	15-ADON	NIV	ZEN
Recovery rate (%) ^1^	96	74	92	99	95
LOD ^2^	0.35	0.45	0.66	0.33	0.03
LOQ ^2^	1.15	1.50	2.21	1.16	0.12

^1^ Expressed as percent recovery of input mycotoxins; ^2^ Expressed in ng·m^−3^.

**Table 2:** Incidence and level of DON, 3-ADON, 15-ADON, NIV and ZEN in aerosols.

Variables	DON	3-ADON	15-ADON	NIV	ZEN
**Overall samples (N = 137)**					
N > LOD ^1^ (%)	123 (90)	17 (12)	12 (9)	123 (90)	106 (77)
N > LOQ ^1^ (%)	48 (35)	3 (2)	1 (1)	61 (44)	106 (77)
Mean concentration ± SD ^2^	35.7 ± 40.8	9.6 ± 7.0	21.8	28.5 ± 41.4	2.4 ± 2.5
Highest concentration	243.9	17.2	21.8	297.2	15.6
**During threshing (N = 78)**					
N > LOD (%)	73 (94)	10 (13)	6 (8)	73 (94)	67 (86)
N > LOQ (%)	33 (42)	3 (4)	1 (1)	41 (53)	67 (86)
Mean concentration ± SD	27.8 ± 42.3	9.6 ± 7.0	21.8	20.0 ± 22.9	1.3 ± 0.7
Highest concentration	243.9	17.2	21.8	107.4	3.8
**During grain unloading (N = 59)**					
N > LOD (%)	50 (85)	7 (12)	6 (10)	50 (85)	39 (66)
N > LOQ (%)	15 (25)	0 (0)	0 (0)	20 (34)	39 (66)
Mean concentration ± SD	53.1 ± 32.4	<LOQ ^3^	<LOQ ^4^	45.9 ± 62.1	4.4 ± 3.3
Highest concentration	121.4	<LOQ ^3^	<LOQ ^4^	297.2	15.6

^1^ LOD: limit of detection; LOQ: limit of quantification; ^2^ Concentrations in ng·m^−3^; ^3^ 1.5 ng·m^−3^; ^4^ 2.2 ng·m^−3^.

**Table 3:** Exposure levels to DON, 3-ADON, 15-ADON, NIV and ZEN during grain handling activities.

Variables	DON	3-ADON	15-ADON	NIV	ZEN
Threshing					
in the cab ^1^ (N = 7)	3.0 ± 1.8	<LOQ ^2^	<LOD ^3^	2.1 ± 1.7	0.2 ± 0.2
N > LOD (%)	7 (100)	1 (14)	0 (0)	5 (71)	4 (57)
Grain unloading					
in the office ^1^ (N = 5)	1.6 ± 0.4	<LOD ^4^	<LOQ ^5^	0.9 ± 1.3	0,08 ± 0.1
N > LOD (%)	5 (100)	0 (0)	1 (20)	2 (40)	2 (40)
in-out ^1^ (N = 6)	6.7 ± 3.0	< LOQ ^2^	< LOD ^3^	3.6 ± 1.7	0.6 ± 0.3
N > LOD (%)	6 (100)	2 (33)	0 (0)	6 (100)	6 (100)
at terminal ^1^ (N = 1)	16.1	4.2	< LOD ^3^	6.6	0.8
N > LOD (%)	1 (100)	1 (100)	0 (0)	1 (100)	1 (100)
Cleaning harvester					
around the machine ^1^ (N = 4)	64.7 ± 79.0	6.2 ± 10.7	<LOD ^3^	58.9 ± 65.9	3.3 ± 2.3
N > LOD (%)	3 (75)	1 (25)	0 (0)	4 (100)	3 (75)

^1^ Concentration ± SD in ng·m^−3^; ^2^ 1.5 ng·m^−3^; ^3^ 0.7 ng·m^−3^; ^4^ 0.5 ng·m^−3^; ^5^ 2.2 ng·m^−3^.

## Supplementary Materials

The following are available online at www.mdpi.com/2072-6651/8/12/370/s1, Table S1: Incidence of DON, 3-ADON, 15-ADON, NIV and ZEN in aerosols per cultivar, Table S2: MS parameters collision cell energies and capillary voltage for each compound.
