# Supplementary material for: Frequent Occupational Exposure to Fusarium Mycotoxins of Workers in the Swiss Grain Industry
Source: Toxins (Basel). 2016 Dec 12;8(12):370. doi: 10.3390/toxins8120370 (PMC5198564; doi:10.3390/toxins8120370)
Supplement: Supplementary file 1 [file toxins-08-00370-s001.docx]

Supplementary Materials: Frequent Occupational Exposure to *Fusarium* Mycotoxins of Workers in the Swiss Grain Industry

Hélène Niculita-Hirzel, Gregoire Hantier, Ferdinand Storti, Gregory Plateel and Thierry Roger

**Table S1.** Incidence of DON, 3-ADON, 15-ADON, NIV and ZEN in aerosols per cultivar.

| **Cultivar** | **Incidence of DON, n (%)** | **Incidence of 3-ADON, n (%)** | **Incidence of 15-ADON, n (%)** | **Incidence of NIV, n (%)** | **Incidence of ZEN, n (%)** |
| --- | --- | --- | --- | --- | --- |
| Apache (N = 1) | 1 (100) | 0 (0) | 0 (0) | 1 (100) | 0 (0) |
| Arina (N = 5) | 5 (100) | 0 (0) | 0 (0) | 5 (100) | 5 (100) |
| Caphorn (N = 1) | 1 (100) | 0 (0) | 0 (0) | 1 (100) | 0 (0) |
| Claro (N = 12) | 9 (75) | 1 (8) | 0 (0) | 9 (75) | 8 (67) |
| Combin (N = 1) | 1 (100) | 0 (0) | 1 (100) | 1 (100) | 1 (100) |
| Forel (N = 8) | 7 (88) | 1 (12.5) | 0 (0) | 7 (88) | 5 (63) |
| Galaxy (N = 4) | 2 (50) | 0 (0) | 0 (0) | 2 (50) | 2 (50) |
| Impression (N = 6) | 6 (100) | 1 (17) | 0 (0) | 6 (100) | 5 (83) |
| Levis (N = 8) | 8 (100) | 0 (0) | 0 (0) | 8 (100) | 8 (100) |
| Ludwig (N = 9) | 9 (100) | 3 (33) | 2 (22) | 9 (100) | 7 (78) |
| Manhattan (N = 5) | 5 (100) | 1 (20) | 0 (0) | 5 (100) | 5 (100) |
| Mulan (N = 1) | 1 (100) | 0 (0) | 0 (0) | 1 (100) | 1 (100) |
| Pollux (N = 1) | 1 (100) | 0 (0) | 0 (0) | 1 (100) | 1 (100) |
| Runal (N = 10) | 10 (100) | 4 (40) | 2 (20) | 10 (100) | 9 (90) |
| Scaro (N = 4) | 4 (100) | 0 (0) | 0 (0) | 4 (100) | 4 (100) |
| Siala (N = 20) | 17 (85) | 2 (10) | 1 (5) | 17 (85) | 12 (60) |
| Soisson (N = 1) | 0 (0) | 0 (0) | 0 (0) | 0 (0) | 0 (0) |
| Spelt wheat (N = 2) | 2 (100) | 0 (0) | 0 (0) | 2 (100) | 2 (100) |
| Tapidor (N = 4) | 4 (100) | 1 (25) | 0 (0) | 4 (100) | 4 (100) |
| Tirone (N = 1) | 1 (100) | 0 (0) | 0 (0) | 1 (100) | 0 (0) |
| Titlis (N = 3) | 3 (100) | 0 (0) | 1 (33) | 3 (100) | 3 (100) |
| Tommy (N = 1) | 1 (100) | 0 (0) | 0 (0) | 1 (100) | 1 (100) |
| Triticale (N = 3) | 3 (100) | 0 (0) | 1 (33) | 3 (100) | 3 (100) |
| Wiva (N = 3) | 3 (100) | 0 (0) | 0 (0) | 3 (100) | 3 (100) |
| Zinal (N = 19) | 16 (84) | 1 (5) | 4 (21) | 16 (84) | 14 (74) |
| Total (N = 137) | 123 (90) | 17 (12) | 12 (9) | 123 (90) | 106 (77) |

**Table S2.** MS parameters collision cell energies and capillary voltage for each compound.

| **Mycotoxins** | **Retention Time (min)** | **Q1 (*m/z*)** | **Q3 (*m/z*)** | **RF Lens (V)** | **CE (V)** |
| --- | --- | --- | --- | --- | --- |
| NIV | 1.79 | 311.1 | 281.1 | 45 | 10.2 |
| ^13^C NIV | 1.79 | 326.2 | 295.1 | 45 | 10.2 |
| DON | 2.65 | 295.1 | 265.1 | 41 | 10.2 |
| ^13^C DON | 2.67 | 310.1 | 279.1 | 41 | 11.3 |
| 3-ADON | 10.42 | 337.1 | 307.1 | 41 | 10.2 |
| ^13^C 3-ADON | 10.43 | 354.2 | 323.1 | 41 | 10.2 |
| 15-ADON | 10.14 | 337.1 | 150.0 | 41 | 27 |
| ZEN | 15.14 | 317.2 | 175.0 | 76 | 23 |
| ^13^C ZEN | 15.13 | 335.2 | 185.0 | 76 | 25 |
